# Effects of cerebellar transcranial direct current stimulation on upper limb motor function after stroke: study protocol for the pilot of a randomized controlled trial

**DOI:** 10.1186/s40814-022-01223-9

**Published:** 2022-12-14

**Authors:** Akiko Yuasa, Shintaro Uehara, Kazuki Ushizawa, Takamichi Toyama, Jose Gomez-Tames, Akimasa Hirata, Yohei Otaka

**Affiliations:** 1 Department of Rehabilitation Medicine I, Fujita Health University School of Medicine, 1-98 Dengakugakubo, Kutsukake-cho, Toyoake, Aichi 470-1192 Japan; 2 Faculty of Rehabilitation, Fujita Health University School of Health Sciences, 1-98 Dengakugakubo, Kutsukake-cho, Toyoake, Aichi 470-1192 Japan; 3 Department of Electrical and Mechanical Engineering, Nagoya Institute of Technology, Gokiso-cho, Showa-ku, Nagoya, Aichi 466-8555 Japan; 4 Center of Biomedical Physics and Information Technology, Nagoya Institute of Technology, Gokiso-cho, Showa-ku, Nagoya, Aichi 466-8555 Japan; 5 Center for Frontier Medical Engineering, Chiba University, 1-33 Yayoi-cho, Inage-ku, 263-8522 Chiba, Japan

**Keywords:** Transcranial direct current stimulation (tDCS), Stroke, Constraint-induced movement therapy (CIMT), Cerebellum

## Abstract

**Background:**

Transcranial direct current stimulation (tDCS) is a technique that can noninvasively modulate neural states in a targeted brain region. As cerebellar activity levels are associated with upper limb motor improvement after stroke, the cerebellum is a plausible target of tDCS. However, the effect of tDCS remains unclear. Here, we designed a pilot study to assess: (1) the feasibility of a study that aims to examine the effects of cerebellar tDCS combined with an intensive rehabilitation approach based on the concept of constraint-induced movement therapy (CIMT) and (2) the preliminary outcome of the combined approach on upper limb motor function in patients with stroke in the chronic stage.

**Methods:**

This pilot study has a double-blind randomized controlled design. Twenty-four chronic stroke patients with mild to moderate levels of upper limb motor impairment will be randomly assigned to an active or sham tDCS group. The participants will receive 20 min of active or sham tDCS to the contralesional cerebellum at the commencement of 4 h of daily intensive training, repeatedly for 5 days per week for 2 weeks. The primary outcomes are recruitment, enrollment, protocol adherence, and retention rates and measures to evaluate the feasibility of the study. The secondary outcome is upper limb motor function which will be evaluated using the Action Research Arm Test, Fugl-Meyer Assessment, for the upper extremity and the Motor Activity Log. Additionally, neurophysiological and neuroanatomical assessments of the cerebellum will be performed using transcranial magnetic stimulation and magnetic resonance imaging. These assessments will be conducted before, at the middle, and after the 2-week intervention, and finally, 1 month after the intervention. Any adverse events that occur during the study will be recorded.

**Discussion:**

Cerebellar tDCS combined with intensive upper limb training may increase the gains of motor improvement when compared to the sham condition. The present study should provide valuable evidence regarding the feasibility of the design and the efficacy of cerebellar tDCS for upper limb motor function in patients with stroke before a future large trial is conducted.

**Trial registration:**

This study has been registered at the Japan Registry of Clinical Trials (jRCTs042200078). Registered 17 December 2020

## Background

Functional recovery of the upper limb is one of the primary goals of those who suffer a stroke. In the last two decades, transcranial direct current stimulation (tDCS) has been widely investigated as a noninvasive brain stimulation technique that can enhance rehabilitation outcomes along with the modulation of brain activity [1]. Several studies using functional magnetic resonance imaging (MRI) [2, 3] or transcranial magnetic stimulation (TMS) [4–7] have shown that tDCS has the potential to enhance neural plasticity. Many studies have attempted to facilitate motor recovery after stroke by applying tDCS to the primary motor cortex (M1) [8, 9]. However, there is no consensus among systematic reviews regarding the effectiveness of tDCS for motor function after stroke. For instance, a recent review suggested that tDCS has significant effects on upper limb motor function in patients with stroke in the chronic stage [8]. However, a network meta-analysis, which synthesized the effects of multiple interventions (i.e., anodal, cathodal, and bilateral tDCS), showed that only cathodal tDCS had moderate effects on levels of activities of daily living (ADL), while no significant effects on the upper limb motor function were noted [9].

Recently, the neuromodulation of the cerebellum has gained increased attention. The cerebellum has multiple connections with motor and non-motor cerebral cortices and plays an important role as a hub of the brain network [10, 11]. Among a wide range of cerebellar functions, its contribution to the motor domain, especially the learning processes, would be essential to patients after stroke [12, 13]. Several imaging studies have suggested that increased cerebellar activity is associated with motor function improvement after stroke [14–18]. On the other hand, cerebellar inactivity such as crossed cerebellar diaschisis, which is caused by decreased blood flow and metabolism in the cerebellar hemisphere due to decreased neural inputs after stroke, is associated with functional impairments [19, 20]. Furthermore, it has been reported that functional connectivity between M1 and the cerebellum [11, 21] and the structural integrity of the cortico-cerebellar tract [17, 18] seem to be related to motor recovery after stroke. These findings demonstrate the importance of the cerebellum and its functional connectivity to motor cortices, indicating that upregulating cerebellar neural activity would be beneficial for motor recovery after stroke [22, 23]. Supporting this hypothesis, animal studies reported that, compared to sham stimulation, applying deep brain stimulation to the cerebellum in stroke-model rats resulted in superior motor improvement in association with neuroplastic changes in the area around a lesion [24–26]. Considering these findings, the cerebellum is a promising target for neuromodulation to facilitate improvements in motor function after stroke.

In stroke rehabilitation, intensive task-oriented training is crucial for facilitating motor recovery. Constraint-induced movement therapy (CIMT) is a well-known approach for improving upper limb motor function after stroke. The key element in CIMT is that it encourages the use of the affected arm through intensive functionally oriented tasks [27, 28]. Although it is generally accepted that neural and functional recovery reaches a plateau around 6 months after stroke onset [29], reviews have shown the effectiveness of CIMT in post-stroke survivors in the chronic stage [30, 31]. In addition, several stroke guidelines highly recommend CIMT as a beneficial approach for improving upper limb motor function and activity especially in the chronic stage [32, 33]. Furthermore, studies reported that CIMT combined with tDCS [34, 35] or combined with tDCS and the peripheral electrical stimulation [36] of motor-related cerebral regions induced greater effects on upper limb motor function compared to CIMT alone.

In view of these, we hypothesized that a combination of cerebellar tDCS and intensive movement therapy would further facilitate the improvement of motor function in stroke patients in the chronic stage. To clarify this hypothesis, we designed the present study to assess (1) the feasibility of a study that will examine the effects of cerebellar tDCS combined with intensive upper limb training and (2) the preliminary effects of this combined approach on upper limb motor function. The present study details a pilot trial that will be conducted before a large-scale randomized controlled trial.

## Methods/design

### Study design

**Fig. 1:**
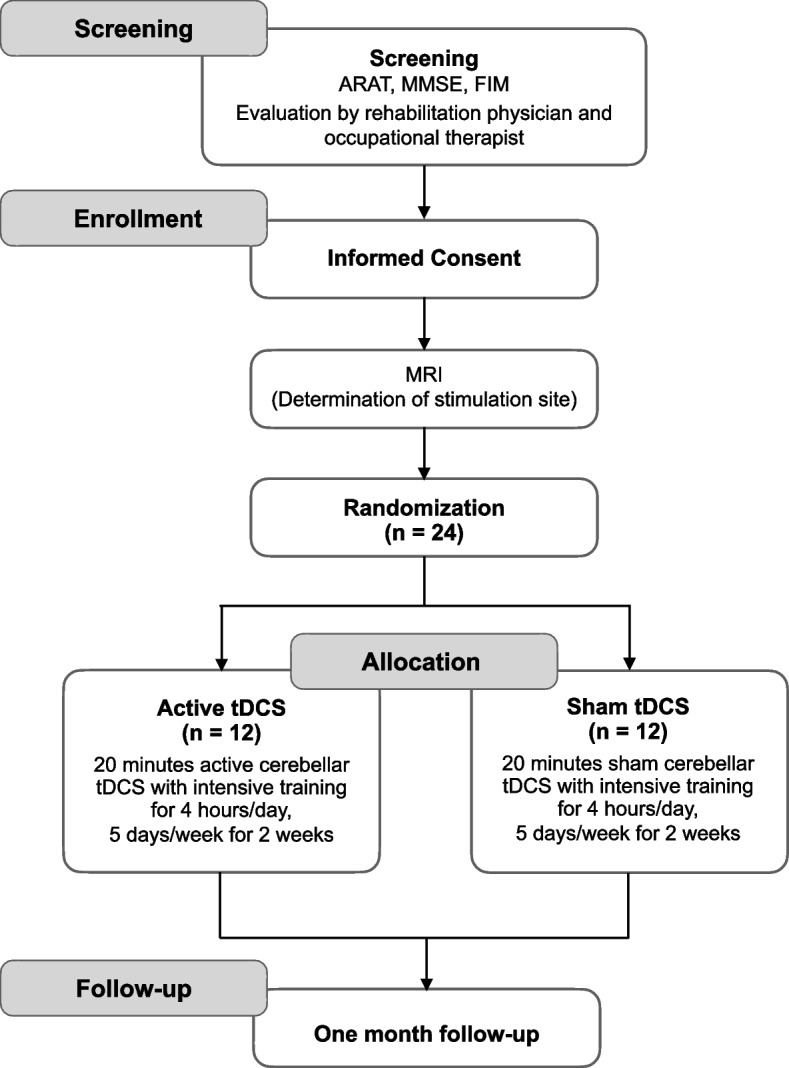
Study flowchart

This study is a double-blind randomized controlled pilot trial and was designed according to the Consolidated Standards of Reporting Trials (CONSORT) extension to pilot trials [37]. The study flowchart is shown in Fig. 1. Twenty-four stroke patients will be enrolled and randomly allocated to an active or sham tDCS group. This study will be conducted at Fujita Health University Hospital, Aichi, Japan. All participants will provide written informed consent before participation according to the Declaration of Helsinki of 1964, as revised in 2013. This study has been approved by the Certified Review Board at Fujita Health University (approval No. CR21-029) and registered at the Japan Registry of Clinical Trials (jRCTs042200078).

### Participants

The inclusion criteria are as follows: (1) age 40–79 years, (2) first-ever unilateral supratentorial ischemic or hemorrhagic stroke, (3) time exceeding 6 months after stroke onset, (4) mild to moderate upper limb hemiparesis [able to score ≥ 1 point on at least one item either in the grasp, grip, or pinch subscale with assessing all items of the ARAT; total score of 10–51], and (5) independent walking ability [score of ≥ 6 on the Functional Independence Measure (FIM) for a walk]. The exclusion criteria are as follows: (1) unstable physical condition, (2) inability to communicate due to severe language impairment, (3) neurological disorders other than stroke, (4) facial sensory deficits, (5) inability to perform training because of psychological disorders or cognitive dysfunction, (6) cognitive impairments [Mini-Mental State Examination (MMSE) < 24] [38, 39], (7) inability to perform training because of musculoskeletal disorders, (8) botulinum toxin injections into the arm within the last 6 months, and (9) any contraindications to tDCS, TMS, and MRI such as a history of epilepsy, metallic implants, cardiac pacemaker, drug or alcohol abuse, or pregnancy.

### Recruitment

Potential participants will be identified from the outpatient clinic of the University Hospital and the local community. The advertisement for the study is open to the public via our department website to allow patients in the local community to access relevant information. Individuals who are interested in this study will be required to make an appointment for screening. The eligibility of potential participants will first be carefully assessed by a physician. They will then be screened for motor and cognitive functions using the ARAT and MMSE by a trained occupational therapist. Written informed consent will be obtained from those who fulfill the criteria and are willing to take part in this study.

### Randomization

Participants will be stratified according to age and ARAT score and randomly allocated to either the active tDCS or sham tDCS group using a computer-generated, permuted block randomization method with permuted block sizes of 2 and 4. The random allocation will be conducted using the Research Electronic Data Capture (REDCap) tools [40] by independent researchers who will not be involved in any intervention and assessments.

### Interventions

**Fig. 2:**
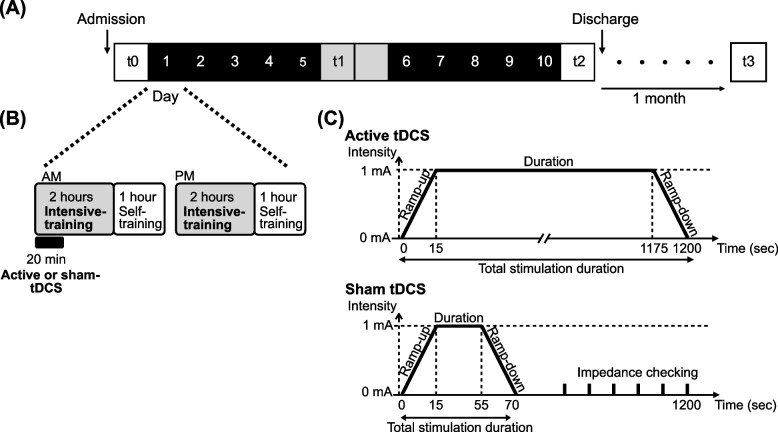
Study protocol. **A** Schedule of the study. The intensive upper limb training and tDCS interventions will be repeated for 5 consecutive days per week for 2 weeks (i.e., 10 days of intervention). During the middle 2 days, the participants will receive only the upper-limb intensive training and self-training. Assessments will be conducted prior to the intervention (t_0_, baseline), at the middle of the intervention (t_1_, middle), immediately after the intervention (t_2_, post), and 1 month after the intervention (t_3_, follow-up). **B** Training schedule per day. Participants will undergo intensive upper limb training for 4 h per day (2 h per half-day) and receive cerebellar tDCS (active or sham) for 20 min during the beginning of the training. They also engage in self-training for 2 h per day (1 h per half-day). **C** Time course of active (upper panel) and sham (bottom panel) tDCS conditions

A time schedule of this study is shown in Fig. 2A. The participants will be admitted to Fujita Health University Hospital. The participants will receive individual therapy of intensive upper limb training for 4 h per day (2 h per half-day) and engage in self-training for 2 h per day (1 h per half-day) and receive cerebellar tDCS (active or sham) for 20 min at the beginning of the intensive training (Fig. 2B), considering that the effects of tDCS on the neural excitability of a targeted brain region last for 30–120 min [2, 5–7]. This will be repeated for 5 consecutive days per week for 2 weeks (i.e., 10 days of intervention). During the intervention period, botulinum toxin injections and any changes of medications that could affect spasticity will not be allowed.

#### tDCS

The stimulation will be delivered using DC STIMULATOR PLUS (NeuroConn, GmbH, Ilmenau, Germany). The anode (5 × 5 cm) will be placed on the cerebellar hemisphere on the contralesional side. Specifically, lobule VI, one of the subregions contributing to motor control of the upper limb [41], will be a focal target of the stimulation. Conventionally, the anode is placed at the fixed position 3 cm lateral to the inion [42]; however, in the present study, the optimal location for targeting the lobule VI will be determined for each individual using computational simulation (see Computational modeling subsection). The cathode (5 × 5 cm) will be placed on the cheek on the contralesional side.

The detailed time course of active and sham tDCS is shown in Fig. 2C. The “study mode” of the NeuroConn stimulator will be used to successfully implement double-blinding using 5-digit codes that activate the active or sham stimulation. Only the independent researchers that conduct the random allocation can access the code list. The experimenter will remain blinded to the information regarding which code belongs to the active or sham stimulation. In the active tDCS group, 1 mA constant current will be delivered for 20 min, whereas in the sham tDCS group, 1 mA constant current will be delivered for 40 s to induce similar scalp sensations. In both groups, the current will be ramped up to 1 mA and ramped down over 15 s at the start and end of the stimulation [43]. During the sham tDCS condition, only a weak current of 110 μA will be delivered every 550 ms after the ramped-down periods to test the electrode impedance. Only the impedance level and stimulation duration will be displayed in both conditions, which facilitates the blinding of the experimenter. The sham procedure is commonly used in tDCS research and at least 30-s active stimulation with a slow ramp up and down are recommended for effective blinding during sham sessions [44]. The participants will not be given any information about stimulation parameters.

#### Intensive upper limb training

Intensive upper limb training based on the concept of CIMT will be provided by five trained occupational therapists who will be blinded to the intervention allocation. One of the occupational therapists with 3 years of CIMT experience trains the others and the training approach will be standardized among them. Each participant will be provided with the intensive upper limb training one-on-one by total 3–4 therapists throughout the intervention period depending on their work shift. CIMT is a therapeutic package that consists of repetitive and task-oriented training, behavioral methods for transferring the use of the affected arm in life situations, and constraining the use of the less affected arm to achieve participants’ specific goals [45]. Since previous studies have reported that physical restraint has no significant effects on the outcome of CIMT [46, 47], no restraints will be applied to the unaffected arm of the participants in this study. Another difference compared to the original CIMT is with respect to the training hours per day, with 4 hours in this study compared to 6 hours in the original [27]. To accomplish sufficient training, participants will be encouraged to use their affected arm in activities of daily living. Additionally, the participants will engage in self-training programmed by the occupational therapists for 2 h per day (Fig. 2B).

At the beginning of the intensive training, specific activities which the participant will aim to be able to reacquire and perform using their affected arm in the ADL/instrumental ADL will be discussed and selected as goals between a therapist and participant. Furthermore, training programs (task-oriented training) specific to an individual participant will be determined by the occupational therapists, considering the goals and remaining function of the affected arm of each participant. For example, when a participant’s goal is to reacquire eating, task-oriented training would be moving blocks to several directions and heights and more practical training of picking up small objects and bringing them to the mouth using a utensil, etc. To provide consistent training among the therapists, they will discuss and share the training programs using videos and photos (types of objects, motion, etc.) throughout the intervention period, at least two times at the beginning and middle (after 1 week) of the intervention period. If necessary, functional electrical stimulation will be provided to enhance muscle contractions and perform functional tasks, but rehabilitative robots will not be allowed.

#### Computational modeling

**Fig. 3:**
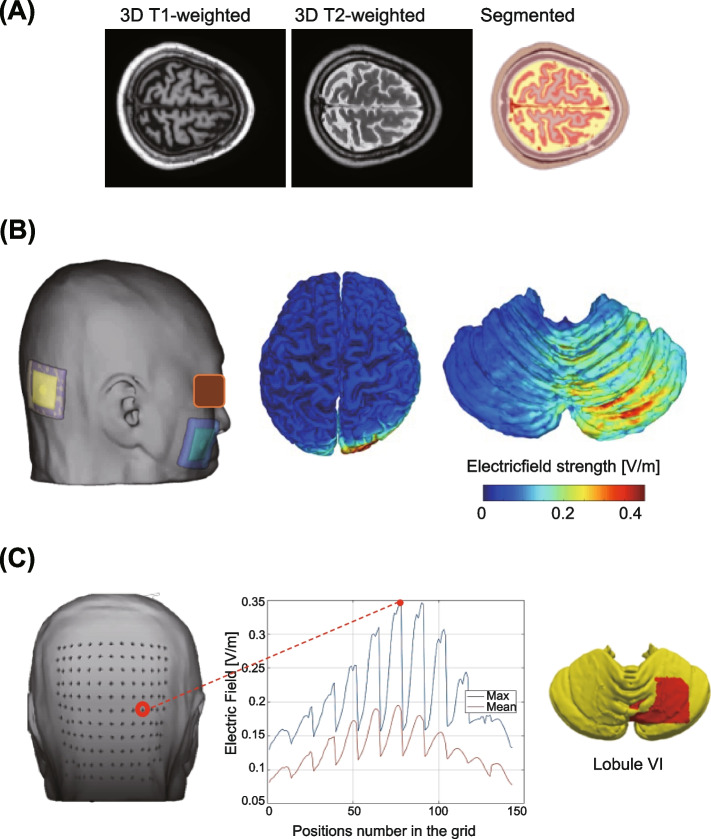
Computational method to determine the optimal individualized position of the tDCS montage. **A** Head model segmentation. The volume conductor of the head model of each participant will be constructed from the segmentation of MRI data. **B** Computational modeling of a representative example. The individualized electric field on the cerebellum using a bipolar montage (anode in yellow and cathode in blue) will be simulated. **C** Optimal montage position. The optimal location of the anode that maximizes the electric field strength (maximum and average) on the region of interest (lobule VI) is selected from a grid of 13 × 11 positions

Figure 3 shows the method that will be used to optimize the location of the tDCS montage at the individual level. First, the head model will be treated as a passive volume conductor constructed from 3D T1- and T2-weighted MRI of each participant before the experiment. The computational method has been reported in detail in a previous report [48, 49]. It involves using the scalar potential finite difference method with fast computational techniques to accelerate the computational assessment before the intervention [50, 51]. The computational model estimates the individualized electric field intensity and distribution on the cerebellar region by applying a bipolar montage throughout the electrodes (5 × 5 cm) with an injection current of 1 mA, as used in the experiment. The cathode will be fixed on the buccinator muscle on the cheek on the contralesional side to avoid high-electric currents flowing on the cerebrum. The anode will be placed on a grid (13 × 11, size of grid = 10 mm) centered on the inion to identify the position that generates the highest electric field strength (maximum and average values), with lobule VI as the target area. The optimal electrode location will be determined based on a reference landmark (10–20 system) and the Montreal Neurological Institute coordinates for confirmation.

### Outcomes

#### Feasibility

Referring to the previous recommendations [52, 53], we will evaluate the feasibility of this pilot study based on the following measures: (1) recruitment and enrollment rates: the proportion of participants who meet the eligibility criteria and provide consent after baseline screening (enrollment rate) and the mean number of participants enrolled in the study per month (recruitment rate); (2) adherence rate: the proportion of completed training hours including individual therapy and self-training of the scheduled hours, which will be used to assess the acceptability of the interventions; and (3) retention rate: the proportion of participants who complete the secondary outcome measurement (i.e., ARAT) from the baseline to follow-up assessments.

These measures will be used as the progression criteria to determine whether to proceed with a future main trial [54]. The criteria are as follows: (1) enrollment rate: more than 30% of screened patients consent to enroll [55]; (2) recruitment rate: more than 0.8 participants are enrolled per month [20% of total target recruitment (5 participants) are enrolled in 6 months]; (3) adherence rate: participants complete more than 90% of scheduled training hours (61 hours on average); and (4) retention rate: more than 80% of enrolled participants (20 participants) with all outcomes until follow-up [30, 56]. If at most three criteria are failed and the issues behind them are manageable, the main trial will proceed with some protocol modifications. If all the criteria are not satisfied, the main trial will not proceed [54, 57]. It should be noted that, although the criteria are determined based on the previous studies and our own experiences, we may need to flexibly reconsider the criterion for the recruitment rate in the case of the COVID-19 pandemic.

#### Safety evaluation

To evaluate the safety of the intervention and overall protocol, we will record adverse events such as burns to the skin, prolonged abnormal cutaneous sensation, dizziness, fatigue, and pain related to overuse of the affected arm. To monitor the existence of the temporary side effects, especially of the tDCS, we will provide participants with a questionnaire [58] after every 20 min of active or sham tDCS intervention. The questionnaire consists of 14 items regarding symptoms which will enable us to determine if the participants are experiencing severe symptoms. The score ranges from 1 to 10, from absent to severe symptoms.

#### Clinical outcomes

**Fig. 4:**
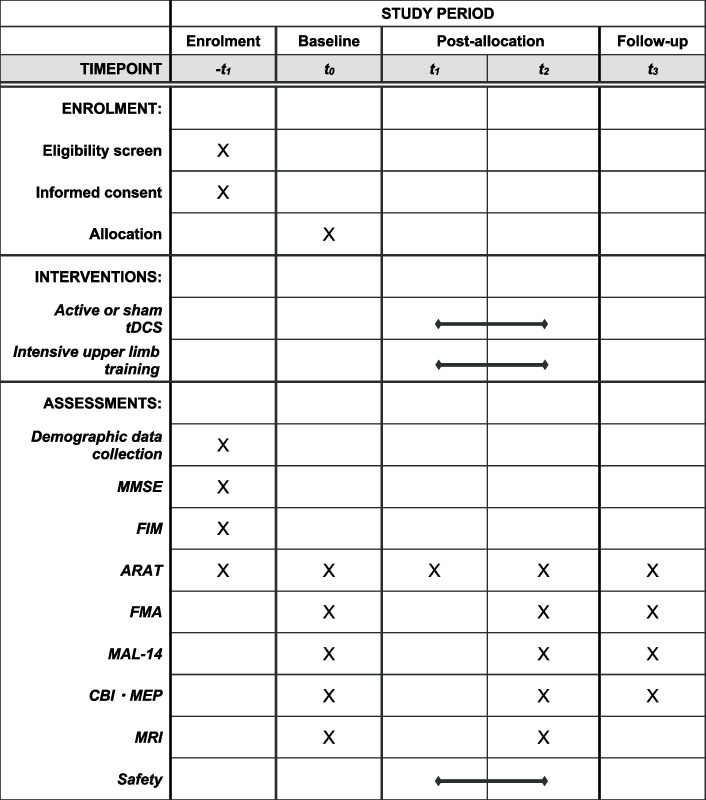
SPIRIT figure. tDCS, transcranial direct current stimulation; MMSE, Mini-Mental State Examination; FIM, functional independence measure; ARAT, Action Research Arm Test; FMA, Fugl-Meyer assessment for the upper extremity; MAL-14, Motor Activity Log-14; CBI, cerebellar brain inhibition; MEP, motor-evoked potential; MRI, magnetic resonance imaging

The schedule of data collection is summarized in SPIRIT figure (Fig. 4). Clinical assessments of the upper limb and neurophysiological and neuroanatomical assessments will be conducted prior to the intervention (baseline, t_0_), at the middle of the intervention [middle, t_1_ (ARAT only)], immediately after the intervention (post, t_2_), and 1 month after the intervention (follow-up, t_3_).

The effects of the interventions will be assessed using ARAT, Fugl-Meyer Assessment (FMA) for the upper extremity, and Motor Activity Log-14 (MAL-14). ARAT is a clinical measurement that consists of 19 tests with 4 subscales: grasp, grip, pinch, and gross movement, which assess upper limb motor function [59, 60]. The quality of movement is scored on an ordinal 4-point scale, ranging from 0 to 3 in each test, with a maximum score of 57 [59]. ARAT is classified as an outcome measurement of activity capacity [61, 62] based on the International Classification of Functioning framework [63], and it shows excellent validity and reliability in chronic stroke patients [64]. FMA is classified as an outcome measurement of body functions [62] based on the International Classification of Functioning framework [63]. FMA for the upper extremity consists of 33 items including reflex testing, movement observation, grasp testing, and coordination assessment. The score ranges from 0 to 66. FMA shows excellent validity and reliability in chronic stroke patients [64, 65]. MAL-14 is a structured interview to assess how much (amount of use: AOU) and how well (quality of movement: QOM) a patient uses the paretic hand and arm during activities of daily living [66, 67]. For the AOU assessment, the score ranges from 0 (never uses the arm) to 5 (uses the arm as often as before the stroke), while for the QOM assessment, the score ranges from 0 (never uses the arm) to 5 (uses the arm as well as before the stroke). MAL-14 is classified as an outcome measurement of activity performance [61, 62] based on the International Classification of Functioning framework [63], and it shows excellent validity and reliability in chronic stroke patients [68].

ARAT and FMA will be video-recorded [69], and video-based assessments will be conducted by two trained occupational therapists who will be blinded to the allocation and will not be involved in the interventions. In addition, the following participant baseline characteristics will be collected: age, the affected side of the brain, time since onset, and FIM and MMSE scores.

#### Neurophysiological assessments

**Fig. 5:**
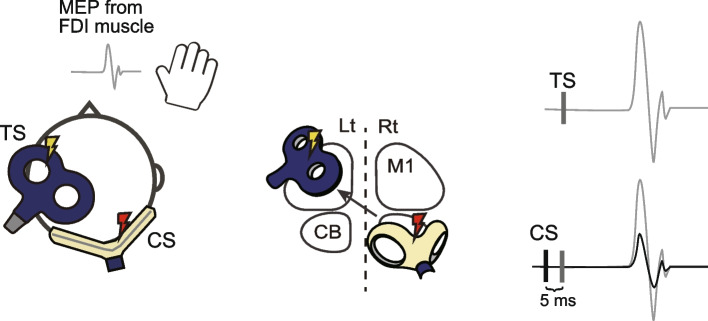
Position of the TMS coils for measuring CBI and MEP. A figure-of-eight coil will be placed over the ipsilesional side of M1 to deliver the test stimuli for eliciting MEP in the FDI muscle. A double-cone coil will be placed over the contralesional side of the cerebellum to deliver the CS. The CS will be delivered 5 ms prior to the TS. CBI will be calculated as the ratio between the peak-to-peak amplitude of the conditioned MEP (black line) and the unconditioned test MEP (grey line). MEP, motor-evoked potential; FDI, first dorsal interosseous; TS, test stimulation; CS, conditioning stimulation; M1, primary motor cortex; CBI, cerebellar brain inhibition; CB, cerebellum

As an index of cerebellar excitability, we will assess the magnitude of cerebellar inhibition (CBI) to the contralateral M1 using a paired-pulse TMS paradigm (Magstim 200^2^, Magstim Company, Whitland, UK) (Fig. 5). CBI will be measured by delivering a conditioning stimulus (CS) over the cerebellum for 5 ms prior to a test stimulus (TS) over M1, resulting in the reduction of motor evoked potential (MEP) elicited by TS over M1 [70]. CBI is thought to be driven by inhibitory outputs from the cerebellar cortex to the deep cerebellar nuclei that have excitatory outputs to the contralateral M1 [71]. Therefore, changes in CBI magnitude can be interpreted as cerebellar excitability changes [72, 73]. We will assess the cerebellar excitability changes with the assumption that cerebellar plasticity may underlie the intervention effects.

The CS will be delivered to the contralesional side of the cerebellum using a double-cone coil which will be centered at the same location determined by the computation modeling, while the TS will be delivered to the ipsilesional side of M1 using the figure-of-eight coil over the optimal stimulation site (“hot spot”) of the first dorsal interosseous (FDI) muscle (Fig. 5). To determine the intensity of the cerebellar CS, we will first test the brainstem active motor threshold for the pyramidal tract by delivering stimuli over the inion using a single pulse with the double-cone coil. The brainstem active motor threshold is defined as the nearest 5% maximum stimulator output (MSO) that elicits MEPs exceeding 50 μV from the FDI muscle in the affected hand in at least 5 of 10 successive stimuli [74]. The cerebellar CS intensity will be set at 5% below the brainstem active motor threshold [70, 75, 76]. If the threshold is not observed below 80% of MSO, 70% of MSO will be used for the cerebellar CS. For the intensity of TS over M1, we will first assess the resting motor threshold (rMT) of the FDI muscle which is determined as the lowest intensity that evokes an MEP amplitude greater than 50 μV in at least 5 of 10 successive stimuli [74]. Thereafter, the TS intensity will be set at 125% of rMT. For the CBI assessment, in a set of 30 TS, 15 TS will be combined with the preceding CS (conditioned TS), while the other 15 TS will be delivered without CS (unconditioned TS). The inter-stimulus interval for TS will be 4–6 s, and the order of conditioned and unconditioned TS will be randomized. The position of the TS over M1 will be tracked using a neuronavigation system (Brainsight, Rogue Research, Montreal, Canada). CBI will be calculated as the ratio of the mean amplitude of conditioned MEP over the unconditioned MEP.

We will also assess M1 excitability by delivering single-pulse TMS over the ipsilesional M1 as a separate measurement set to confirm that potential CBI changes will not be accompanied by M1 excitability changes. For this purpose, 15 stimuli with an intensity of 125% of rMT will be applied with the inter-stimulus interval of 4–6 s. The mean amplitude will be used as a proxy for M1 excitability.

Surface electromyography will be recorded using a biosignal recording system (Nuropack X1 MEB-2312; Nihon Kohden Corporation, Tokyo, Japan) at the frequency of 5 kHz, with a bandpass filter of 10 Hz to 10 kHz. Recorded analog data will be digitized with a micro 1401 AD converter (Cambridge Electronic Design, Cambridge, UK) and stored on a computer (Signal Software, Cambridge Electronic Design, Cambridge, UK) for offline analysis.

#### MRI

To identify structural characteristics of the individual brain, MR images will be acquired prior to the intervention (baseline, t_0_) and after the intervention (post, t_2_). The brain MRI will be conducted using a 3-T scanner (Vantage Centurian 3T; Canon Medical Systems, Tochigi, Japan). For brain tissue segmentation, 3D T1- and T2-weighted images with high resolution (voxel size: 0.8 × 0.8 × 0.8 mm^3^) will be acquired [77]. In addition, to identify functional and structural connectivity between widespread brain areas, resting-state functional MRI, and diffusion tensor imaging will be obtained [78]. The 3D T1- and T2-weighted images will be used for the computational modeling (see Computational Modeling subsection).

#### Sample size

A previous study proposed the rules of thumb for estimating the sample size of a pilot study based on the anticipated effect size of the future main trial [79]. According to the proposal, if the future main trial is designed around medium to large effect with 80% power and a two-sided 5% significance, a sample size for the pilot trial is set at 10 in each group [79]. Allowing for a 20% of drop-out rate, we will recruit 12 participants in each group.

#### Blinding

Participants, assessors, occupational therapists providing intensive upper limb training, experimenter applying the tDCS, and a researcher analyzing the data will be all blinded to group allocation. Independent researchers who will conduct the random allocation will not be blinded. A researcher who is in charge of patients’ safety, and risk management can access the group allocation via the REDCap if necessary. To quantify the success of blinding, we will ask participants which group they think they were allocated after the intervention period.

#### Analytical methods

The feasibility outcomes will be reported with descriptive statistics. Recruitment, enrollment, adherence, and retention rates will be calculated with a 95% confidential interval. Furthermore, adverse events and their frequency will be recorded. If there are some exclusions or dropouts, the reasons and the step at which they occur will be recorded. For the potential side effects of tDCS, the median score for every 14 items of the questionnaire regarding symptoms will be calculated. Regarding the success of blinding, the percentage of correct guesses will be reported.

The active and sham tDCS groups will be compared with regard to demographic variables to assess group differences using the Student’s *t* test or Mann-Whitney *U* test for continuous variables and the *χ*^2^ test for categorical variables. Group differences of the ARAT, FMA, and MAL-14 scores and neurophysiological measures outcomes will be analyzed using multivariable regression analyses and compared at each assessment time point. To evaluate the efficacy of the present pilot study, an effect size of the ARAT score will be computed, which will be used to determine the sample size for the future main trial.

## Discussion

Neuromodulatory intervention for the cerebellum has been suggested to improve the motor function of stroke survivors in the chronic stage. Our pilot trial aims to assess the feasibility of the proposed study and the efficacy of the application of tDCS to the cerebellum on upper limb motor function in patients after stroke. The findings will contribute to deciding whether to proceed with the future main trial and, if needed, redesigning the protocol such as eligibility criteria, the dose of interventions, assessment schedule, and sample size.

The present study has some potential limitations. First, the study aims to enroll patients with mild to moderate hemiparesis since intensive upper limb training requires voluntary movements of the affected hand to some extent. Therefore, the findings need to be carefully translated into clinical practice especially when considering the intervention effects in severe cases. Second, in conventional cerebellar tDCS, the anode is placed at a fixed position 3-cm lateral to the inion [42], whereas in the present study, the optimal electrode location will be determined for each individual using computational simulation. However, it remains unclear whether this personalized tDCS maneuver will improve outcomes regarding upper limb motor function compared to conventional tDCS. This question should be addressed in another future study.

To our knowledge, this is the first series of studies to evaluate the effects of cerebellar tDCS combined with intensive training on upper limb motor function in chronic stroke patients. We believe that cerebellar tDCS will improve the effect of intensive upper limb training. The comprehensive results from the present study and the forthcoming large-scale trial should provide significant evidence regarding cerebellar tDCS to determine whether it can be a beneficial intervention for stroke patients.

## Data Availability

The datasets used and/or analyzed during the current study are available from the corresponding author on reasonable request.

## Abbreviations

ADL: Activity of daily living
AOU: Amount of use
ARAT: Action Research Arm Test
CBI: Cerebellar brain inhibition
CIMT: Constraint-induced movement therapy
CS: Conditioning stimulus
FDI: First dorsal interosseous
FIM: Functional independence measure
FMA: Fugl-Meyer assessment for the upper extremity
M1: Primary motor cortex
MAL-14: Motor Activity Log-14
MEP: Motor-evoked potential
MMSE: Mini-Mental State Examination
MR/MRI: Magnetic resonance/magnetic resonance imaging
MSO: Maximum stimulator output
QOM: Quality of movement
tDCS: Transcranial direct current stimulation
TMS: Transcranial magnetic stimulation
TS: Test stimulus
